# More Medical Journals

**Published:** 1856-02

**Authors:** 


*More Medical Journals.*

We have received the first two numbers, (January and February,) of the "*Cincinnati Medical Observer;*" a monthly journal of 48 pages, edited by GEORGE B. MENDENHALL, M.D., JOHN A. MURPHY, M.D., and EDWARD B. STEVENS, M.D. The two first named are Professors in the Miami Medical College, and the last is the *publisher*, to whom all communications should be addressed. The number of the journal for February contains a very fair engraved likeness of the late Dr. DANIEL DRAKE.

